# The Cirencester Experiment

**Published:** 1921-06-11

**Authors:** 


### The Cirencester Experiment.

### A PAYING HOSPITAL FOR ALL CLASSES AND ALL INCOMES.

The article under the above title in our issue of April 30 has excited a good deal of interest and brought us inquiries from cottage-hospital managers desiring to put a similar experiment into practice. We therefore, through the courtesy of the Honorary Secretary of the Cirencester Memorial Hospital, Mr. Louis C. Wrigley, give the following additional information in the hope that it may assist others who are confronted with similar problems in their locality:

The main point of the scheme is that every patient should pay something, and the amount of their payment is carefully assessed by the House Committee at their weekly meetings. For the very deserving cases the minimum at Cirencester is 3s. 6d. a week, or 6d. a day; in the case of a man in good employment, it varies according to the number of his family and the circumstances in each case, but may go as high as 3s. 6d. per day. The minimum for private patients is £3 3s. a week, but may go to £10 10s. or £12 12s. In severe cases, if an extra night nurse is required, this is at the cost of the private patient, who also pays for all his medicines or extra luxuries. The Committee contains representatives of the Free Church Council and of the Trade and Labour Council. It carries the confidence of all sections, and friendly relations exist between it and the working men, whose committee organises yearly a carnival, which realises over £200. During the past five years there is no record of an assessment having been disputed or not paid.

The benefit to the hospital of the private patients is obvious, for by means of the income thus provided (£600 in 1920) a higher standard is possible of nursing, food, and service by which the poorer patients benefit. The arrangement has been also found of benefit to the local medical staff, and is looked upon as a small return for their gratuitous work. But the principal feature is the benefit to the middle classes, who, while not wishing to accept the ordinary form of hospital ticket, are often unable to face the tremendous expense of a nursing home or of an operation in their own home. The accommodation at Cirencester is twenty-four beds and four cots; four of these beds are in private rooms for private patients, but in case of urgency private patients may occasionally be accommodated in a four-bed ward, where, although not having a room to themselves, they have to pay the same fee.

